# CAE-ResVGG FusionNet: A Feature Extraction Framework Integrating Convolutional Autoencoders and Transfer Learning for Immature White Blood Cells in Acute Myeloid Leukemia

**DOI:** 10.1016/j.heliyon.2024.e37745

**Published:** 2024-09-12

**Authors:** Tusneem Elhassan, Ahmed Hamza Osman, Mohd Shafry Mohd Rahim, Siti Zaiton Mohd Hashim, Abdulalem Ali, Esmaeil Elhassan, Yusra Elkamali, Mahmoud Aljurf

**Affiliations:** 1 Cancer Center of Excellence, King Faisal Specialist Hospital and Research Center, Riyadh 11564, Saudi Arabia; 2 Department of Information Systems, Faculty of Computing and Information Technology, King Abdulaziz University Rabigh, Saudi Arabia; 3 School of Computing, Universiti Teknologi Malaysia, Johor Bahru 81310, Johor, Malaysia; 4 Faculty of Computer & Information Technology, Sohar University, Sohar, Oman; 5 Institute of Computer Science and Digital Innovation, UCSI University, Federal Territory of Kuala Lumpur; 6 Faculty of mathematical science, university of Khartoum, Sudan; 7 Dept of Hematology, Stem Cell Transplantation and Cellular Therapy, King Faisal Specialist Hospital & Research Centre, Riyadh, Saudi Arabia

**Keywords:** Acute myeloid leukemia, Immature white blood cells, Autoencoder, VGG19, ResNet, Computer-aided diagnosis model, blood smear microscopic images, image classification, Cancer

## Abstract

Acute myeloid leukemia (AML) is a highly aggressive cancer form that affects myeloid cells, leading to the excessive growth of immature white blood cells (WBCs) in both bone marrow and peripheral blood. Timely AML detection is crucial for effective treatment and patient well-being. Currently, AML diagnosis relies on the manual recognition of immature WBCs through peripheral blood smear analysis, which is time-consuming, prone to errors, and subject to inter-observers’ variation. This study aimed to develop a computer-aided diagnostic framework for AML, called "CAE-ResVGG FusionNet", that precisely identifies and classifies immature WBCs into their respective subtypes. The proposed framework leverages an integrated approach, by combining a convolutional autoencoder (CAE) with finely tuned adaptations of the VGG19 and ResNet50 architectures to extract features from CAE-derived embeddings. The process begins with a binary classification model distinguishing between mature and immature WBCs followed by a multiclassifier further classifying immature cells into four subtypes: myeloblasts, monoblasts, erythroblasts, and promyelocytes. The CAE-ResVGG FusionNet workflow comprises four primary stages, including data preprocessing, feature extraction, classification, and validation. The preprocessing phase involves applying data augmentation methods using geometric transformations and synthetic image generation using the CAE to address imbalance in the WBC distribution. Feature extraction involves image embedding and transfer learning, where CAE-derived image representations are used by a custom integrated model of VGG19 and ResNet50 pretrained models. The classification phase employs a weighted ensemble approach that leverages VGG19 and ResNet50, where the optimal weighting parameters are selected using a grid search. The model performance was assessed during the validation phase using the overall accuracy, precision, and sensitivity, while the area under the receiver characteristic curve (AUC) was used to evaluate the model’s discriminatory capability. The proposed framework exhibited notable results, achieving an average accuracy of 99.9%, sensitivity of 91.7%, and precision of 98.8%. The model demonstrated exceptional discriminatory ability, as evidenced by an AUC of 99.6%. Significantly, the proposed system outperformed previous methods, indicating its superior diagnostic ability.

## 1 Introduction

Manual classification of acute myeloid leukemia (AML) cells is time-consuming as well as susceptible to errors and intra- and inter-observer variabilities 1, 2, 3. The classical method for diagnosing leukemia is based on repeated complete blood counts and bone marrow examinations following the observation of symptoms. Blood tests do not always reveal leukemia, especially in the early stages of the disease. In such cases, a bone marrow biopsy is required for further investigation. Manual diagnosis of acute leukemia is subject to human error and increases the likelihood of a false-negative results; either of these outcomes can lead to death due to the rapid progression of this disease. Expert pathologists or laboratory scientists are more likely to successfully detect and classify leukemia; however, previous studies have found that only 76.6% of pathologists agreed on cases involving the diagnosis of leukemia [4]. The manual process of counting cells and diagnosing patients is time-consuming and tedious. Therefore, an automatic cost-effective diagnostic tool must be developed to provide rapid and accurate diagnosis of leukemia.

Deep learning (DL) classification and pattern recognition models have been widely used for image classification across diverse sectors including military services, telecommunications, agriculture, and traffic detection, with notable advancements documented in the literature 5, 6, 7, 8, 9, 10. In medical applications, DL has been effectively employed in various domains such as brain tumor classification, breast cancer detection, hypothyroidism prediction, retinopathy classification, lung cancer detection, and pneumonia classification 11, 12, 13, 14, 15, 16, 17, 18, 19. The integration of artificial intelligence (AI), particularly deep learning methodologies, has significantly transformed the disease detection and classification in the dynamic field of medical diagnostics. AML, a critical hematologic malignancy, is characterized by the rapid proliferation of abnormal white blood cells (WBCs) within the bone marrow. Accurate and rapid identification of immature WBCs is of paramount importance, forming the cornerstone of early diagnosis and formulation of effective treatment strategies.

AML presents a formidable challenge in the field of hematology. The disease’s hallmark is the rapid proliferation of immature blood cells, leading to the replacement of healthy cells and hindering the vital function of bone marrow in the production of essential blood components [20]. A key component of diagnosing acute myeloid leukemia (AML) involves the complex process of differentiating and classifying immature white blood cells (WBCs) into distinct subtypes based on subtle distinguishing features. . The major problem stems from the significant similarities and complex distinctions among these immature cells. In particular, cells in subsequent maturation phases, such as myelocytes and metamyelocytes presents challenges owing to subtle discrepancies and intricate maturation processes 3, 21, 22. The lack of precise criteria for differentiating WBCs at different stages of development adds complexity to this task considering the complicated nature of the growth of these cells 2, 23. Compounding these challenges is the low frequency of specific WBC forms in AML blood samples, confounding machine learning (ML) models’ ability to discern significant features for differentiation^24^, ^25^. Manual identification of immature cells, a process often undertaken by pathologists, is not only laborious and time-consuming but also susceptible to inconsistencies due to intraclass and interclass variations. Moreover, certain advanced microscopes, such as Cellavision DM96, rely on quantitative methods that diminish their sensitivity to blast cells, which are crucial indicators for leukemia diagnosis 25, 26.

In response to these significant challenges, automated solutions incorporating computer vision, alongside traditional machine learning (ML) and advanced deep learning (DL) techniques, have been developed. Traditional ML lies on manual, low-level handcrafted features, while DL extracts abstract, automated features. However, handcrafted features, because of their manual nature and the need for human-defined criteria, present limitations that require specialized expertise.

Research on the feature extraction and classification of immature WBCs varies in their approach to feature extraction, with some employing hand-crafted features such as texture, geometric, color, morphological, and fractal characteristics 27, 28, 29, 30, 31, 32, 33, 34, while others utilize deep learning-based features 3, 25, 35, 36. Additionally, the studies also vary in their scalability and adaptability to different datasets. A key factor affecting generalizability is whether studies use local datasets, collected from specific hospitals, or public datasets, accessible to the broader research community. While many studies utilize local datasets 27, 28, 29, 30, 31, 32, only one public dataset exists for morphological classification of immature WBCs, which has been used in several studies.^3^, ^25^, ^33^, ^34^, ^35^, ^36^. Furthermore, the generalizability of these models is affected by the type of images used in the feature extraction process, particularly between whole images and segmented images. Segmented images, which exclusively depict white blood cells (WBCs) while excluding other blood components or artifacts, offer a more general and context-free learning environment 33, 34. While segmentation has traditionally been important for hand-crafted feature extraction, Elhassan et al. [25] demonstrated that incorporating segmentation significantly enhances model generalization in deep learning-based approaches. However, only a limited number of studies have utilized WBC segmentation within deep learning frameworks, with 25, 35, 36 showing improved results.

The driving motivation of our study was to develop a diagnostic framework that could overcome these limitations and can accurately classify immature WBCs into their distinct subtypes. Our methodology uses a convolutional autoencoder (CAE), which is a specialized neural network architecture for feature extraction. This architecture effectively captures subtle variations in cell shape and structure, thereby enabling accurate differentiation between normal and pathological WBCs. Furthermore, our application of transfer-learning ensemble ensures the adaptability and usefulness of our model on different datasets, providing a solid foundation for a more efficient diagnostic approaches. The contributions of this study are as follows:

1. We have developed a new computer-aided diagnostic framework, referred to as "CAE-ResVGG FusionNet" for classifying immature WBCs.
2. We introduced a newfeature extraction method by integrating CAE-derived image embeddings with integrated fine-tuned VGG19 and ResNet50 pretrained models.
3. We improved the classification accuracy of immature WBCs using a weighted ensemble model of VGG19 and resNet50.

The remainder of this paper is organized as follows: Section 2 summarizes previous research on the same topic. Section 3 explores the dataset and research methodologies. Section 4 presents the experimental results and a discussion. Section 5 summarizes the findings of this investigation.

## 2 Related work

The classification of immature WBCs has not been extensively studied. Current research into AML classification is considered to be of low accuracy. This finding can be attributed to various factors, including the biological complexity of WBCs such as cell density, color, texture, and shape. In addition, immature WBCs in the intermediate stages are commonly misclassified, particularly those in the subsequent stages of hematopoiesis, because of the significant similarities between these types of cells 3, 37, 38, 39. Furthermore, the skewed distribution of WBCs in blood samples leads to imbalanced data, resulting in reduced accuracy when identifying less-common subtypes of immature WBCs 3, 40.

In addition, the process of localizing and segregating WBCs from other components of the blood is challenging and hindered by the overlapping textural similarities of WBCs and irregular boundaries 24, 39. Contemporary AML research is also hampered by the limited number of publicly available experimental datasets. These limitations underscore the need for further research and improved methods for immature WBCs classification.

Previous research in AML can be categorized into two types: research that focuses on DL feature extraction and research based on handcrafted feature extraction to classify immature WBCs into different subtypes.

Several studies have used handcrafted features to classify immature WBCs. Dasariraju et al.[33] used 16 morphological and color features to classify WBCs into four distinct subtypes, achieving an overall accuracy rate of 93%. Dincic et al.[41] used morphological, fractal, and textural characteristics to categorize white blood cells into 11 distinct types, including both mature and immature cells. Their statistical analysis revealed eight key features, which were then applied using a support vector machine algorithm, achieving an average accuracy of 80%.

However, only few studies have employed DL methods to classify immature WBCs. In their research, Qin et al.[42] classified WBCs into 40 groups, including immature WBCs. The overall classification accuracy ranged from 37% to 89%. Matek et al.[3] employed convolutional neural network (CNN) to extract DL features and to classify WBCs into 15 different subgroups. Elhassan et al.[43] proposed an innovative method for feature extraction by combining image processing techniques with deep learning methodology. The methodology involves two consecutive stages: identifying a region of interest (ROI) using the CMYK moment localization technique and extracting DL-based features using a CNN-based feature fusion approach. The extracted features were then used for semantic segmentation. They achieved classification accuracy of 97.57%. Ín another study, Elhassan et al. [22] developed a hybrid multiclassification method that accurately classified atypical WBCs into eight distinct subgroups. The model exhibited an average accuracy rate of 97%, sensitivity rate of 97%, and precision rate of 98%. Bairaboina et al. [36] introduced a model to classify immature WBCs into 12 distinct subtypes, encompassing preprocessing, segmentation via a W-Net based model, feature extraction using a GhostNet-based model, and classification using an optimized ResNeXt-based framework, with hyperparameter optimization achieved using the WHO algorithm. This model demonstrated an accuracy of 98.61%.

## 3 Methodology

### 3.1 Dataset

This study employed a dataset of single-cell morphology (AML Cytomorphology LMU) consisting of leukocytes from both AML patients and non-malignant controls. The dataset comprised 18,365 single-cell images, which were expertly identified and obtained from peripheral blood smears of 100 AML patients and 100 controls. The data were collected at Munich University Hospital between 2014 and 2017. The dataset was classified into 15 distinct types based on individual cell images. Of the total, four cells were leukemia, and the remaining 11 were healthy WBCs. Of the 11 WBCs, seven were mature leukocytes, and the remaining four were immature. Experienced pathologists assessed cancerous and non-cancerous WBCs using a well-established system on morphological classification[44]. The dataset is anonymized to protect privacy and adheres to relevant data protection regulations. It has been employed in four previous studies, each utilizing distinct feature extraction and classification algorithms. The generally comparable results across these studies highlight the dataset's stability and generalizability. However, variations in preprocessing, feature extraction, or classification methods led to improved performance in some studies.

### 3.2 Proposed model

**Figure 1:**
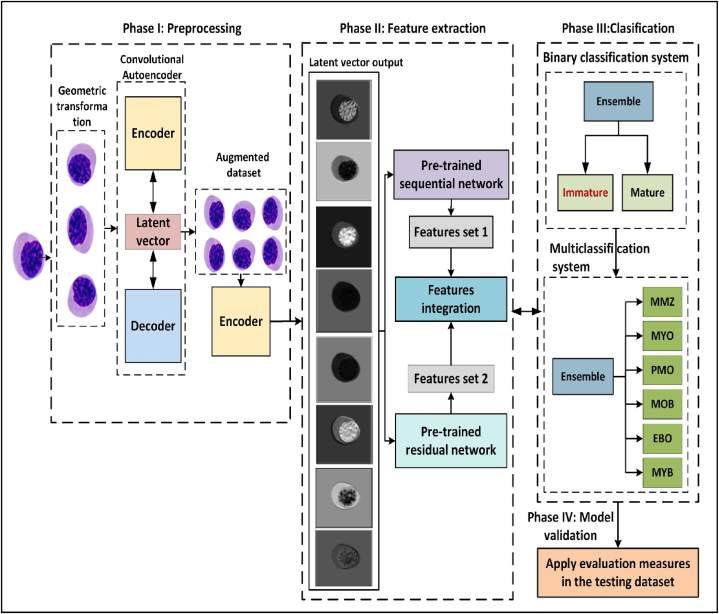
operational framework.

The CAE-ResVGG FusionNet framework follows four distinct phases: preprocessing, feature extraction, classification, and validation. In the preprocessing phase, data augmentation techniques are used to address the imbalance distribution of WBCs, including geometric transformation and synthetic image generation using a CAE [22]. The feature extraction phase involves image embedding and transfer learning, where the new image representations obtained by the CAE are used by two pretrained models: VGG19 and ResNet50. The classification phase employs a weighted ensemble of VGG19 and ResNet50, with the optimal weights determined via a grid search. The model’s performance was evaluated using the overall accuracy, precision, and sensitivity during the validation phase. The area under the receiver characteristic curve (AUC) was used to assess the model’s discriminatory capability (Figure1). The following gives a detailed explanation of each phase.

• **Phase I: Preprocessing**

The initial preprocessing stage involves applying geometric transformations to the segmented images and generating new synthetic images. Segmentation was performed using CMYK-moment localization and a CNN feature fusion model, which was developed by Elhassan et al. [25]. This procedure was performed to address the imbalanced distribution of immature WBCs. The geometric transformation involves rotation, vertical, and horizontal flipping. In addition, CAE were used to generate new synthetic images [45]. The CAE is a neural network architecture consisting of three components, namely the encoder, decoder, and latent vector. The encoder unit applies several convolutional operations to accurately transform the input image into a compressed low-dimensional image. The latent vector stores the compressed low-dimensional image obtained from the encoder. The decoder unit uses low-dimensional image representations (image embeddings) and reverse mapping to reconstruct the input image. The CAE employs unsupervised learning to identify optimal filters that extract the significant features necessary for reconstructing the input images. These filters are then used to transform the input image into a lower-dimensional representation for image compression. The latent representation of the $i^{th}$ feature map can be expressed mathematically as follows:

(1) $$L^{i}=A\;\left(x\;⨂\;w^{i}+b^{i}\right)$$

where, *x* is the input image, $L^{i}$ denotes the latent vector representation, *A* denotes the activation function, ⨂ denotes the 2D convolution operation, $w^{i}$ is the weights, and $b^{i}$ is the bias.

Subsequently, the process involves restoring the output by employing the inverse operation to obtain the original input image. The reconstruction of the input image is represented by the following equation:

(2) $$y=A\sum_{i\epsilon \Omega }\left(L^{i}\;⨂\;{\overset{`}{w}}^{i}+c\right)$$

where y is the reconstructed image, Ω is the set of latent features maps, and c is a constant.

The quality of reconstructed image is then evaluated using mean square error as follows:

(3) $$E=\frac{1}{n}\sum_{i=1}^{n}{\left(x_{i}-y_{i}\right)}^{2}$$

where, *E* is the reconstruction error, *n* is the number of input images, $x_{i}$ is the $i^{th}$ input image, and $y_{i}$ is the $i^{th}$ the reconstructed image.

The optimal parameters are determined by applying the backpropagation algorithm to calculate the gradient of the error function with respect to the weights, which can be expressed asfollows:

(4) $$\frac{\partial E}{\partial W}=x*\;\delta L^{i\;}+{\overset{`}{L}}^{i}*\delta y$$

where $\delta L^{i\;}$ and δy, represent the changes in the latent vector and reconstruction, respectively. The updated weighted values are backpropagated in the network using the stochastic descent algorithm.

**Phase II: Feature extraction**

The feature extraction phase involves the derivation of DL features by applying image embeddings, produced by CAE, to an integrated framework of pretrained models, namely, VGG19 and ResNet50. This comprehensive approach comprises of four fundamental parts:

1. Acquisition of compressed, feature-centric image embeddings using the CAE, to establish a foundational representation of the input data;
2. Incorporation of the CAE-derived embeddings into the convolutional framework of VGG19, a well-known model recognized for its deep network that can effectively capture complex image features;
3. Incorporation the CAE-derived embeddings into the convolutional framework of ResNet50, which combines convolutional processes with shortcut connections, to enable more effective training of deep networks without the typical problems of gradient vanishing or explosion; and
4. Integration of features obtained by VGG19 and ResNet50 to obtain the final feature set.

This layered feature extraction methodology capitalizes on the initial embeddings from the CAE, which are progressively enhanced through the advanced convolutional capabilities of VGG19 and the architectural innovations of ResNet50, enabling a comprehensive and nuanced analysis of image data.

a. Convolutional autoencoder (CAE)

**Figure 2:**
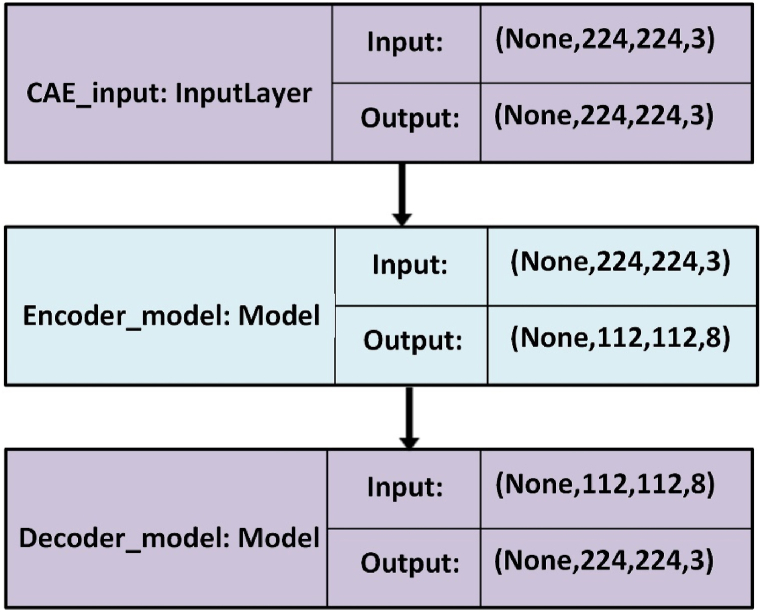
CAE architecture

CAEs are a type of unsupervised CNN that are trained to acquire filters that extract essential features for reconstructing the input images. In this study, the encoder comprised one input layer and three convolutional layers. The input layer consists of 224 × 224 × 3 dimensions, and the three convolutional layers consist of 112 × 112 × 4, 112 × 112 × 6, and 112 × 112 × 8 dimensions. The decoder unit consisted of three convolutional layers (112 × 112 × 8, 112 × 112 × 6, and 112 × 112 × 4) and an output layer (112 × 112 × 3). The CAE architecture is illustrated in Figure 2.

The model used a corpus of 2,711 trainable parameters:1,358 and 1,353 parameters for the encoder and decoder; respectively. The following equations are employed to compute the total number of learned parameters:

(5) S=m∗n∗c

where, *S* is the shape, *m* is the kernel width, *n* is the kernel hight, and *c* is the number of channels.

(6) $$P=\left(\left(m*n*\;f_{Previous}\right)+1\right)*\;f_{Current}$$

where, *P* is the number of parameters, *m* is the kernel width, *n* is the kernel height, $f_{Previous}$ is the number of filters in the previous layer, and $f_{Current}$ is the number of filters in the current layer, and *1* is the bias.

Tables 1 and 2 present the shape, size, and number of parameters for the encoder and decoder units using equations (5), (6).

b. VGGNets

**Table 1:** Encoder

Layers	Filter size	stride	Activation shape	Activation size	Number of parameters
Input layers	-	-	224x224x3	150,528	0
Cov1	3	2	112x112x4	50,176	112
Cov2	3	1	112x112x6	75,264	222
Cov3	3	1	112x112x8	100,352	440
output layer	3	1	112x112x8	100,352	584

**Table 2:** decoder

Layers	Filter size	Upsampling	Activation shape	Activation size	Number of parameters
Input layers	-	-	112x112x8	100,352	0
Cov1	3	2	224x224x8	401,408	584
Cov2	3	-	224x224x6	301,056	438
Cov3	3	-	224x224x4	200,704	220
output layer	3	-	224x224x3	150,528	111

**Figure 3:**
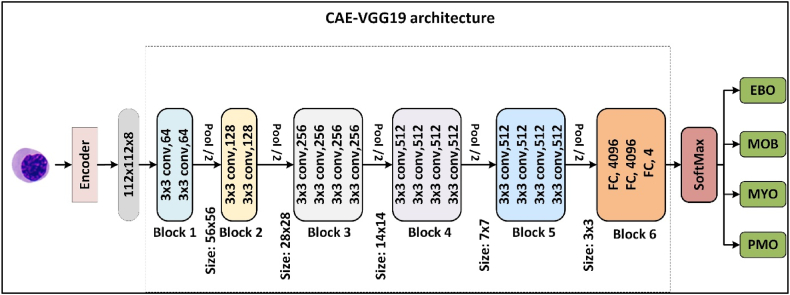
AE-VGG19 architecture.

The VGGNet architecture is a complex CNN framework that was specifically designed to investigate the correlation between CNN depth and classification accuracy. The VGG19 model, a derivative of the VGGNet family, is composed of multiple convolutional blocks followed by fully connected layers [46]. In this study, a highly refined and tailored version of VGG19 was used to extract features from image embeddings. These embeddings have a non-standard shape of 112 × 112 × 8, which differs from the conventional input configuration of (224, 224, 3) for VGG19 (Figure 3).

**Figure 4:**
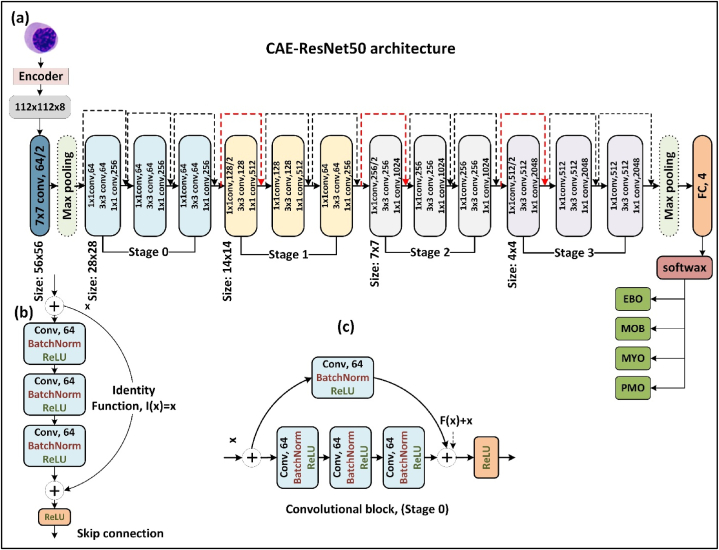
CAE-ResNet50 architecture.

The following is a comprehensive illustration of the VGG19 architecture used in this study:

• **Block 1** comprises two layers containing 3 × 3 convolutional kernels, a stride of 1, and 64 filters. The dimensions of the produced image are 112 × 112 × 8 × 64. Then, a Max-pooling layer with dimensions of 2 × 2 and a stride of 2 is applied. The generated image has dimensions of 56 × 56 × 8 × 64.
• **Block 2** comprises two layers with 3 × 3 convolutional kernels, a stride of 1, and 128 filters. This is followed by a 2 × 2 Max-pooling layer with a stride of 2. The dimensions of the generated image are 28 × 28 × 64 × 128.
• **Block 3** comprises four layers that employ 3 × 3 convolutional layers with a stride of 1 and 256 filters. Subsequently, a 2 × 2 Max-pooling layer is applied. The dimensions of the produced image are 14 × 14 × 128 × 256.
• **Block 4** comprises four layers using a convolutional kernel of size 3 × 3, a stride of 1, and 512 filters, followed by a Max-pooling layer of size 2 × 2 and a stride of 2. The output image is of size 7 × 7 × 256 × 512.
• **Block 5** comprises four layers that employ a 3 × 3 convolutional kernels with a stride of 1 and 512 filters, followed by a Max-pooling layer with a size of 2 × 2 and stride of 2. The output is a 3 × 3 × 512 × 512 image.
• **Block 6** comprises three fully connected layers with 4,096, 4,096, and 4 neurons. The last fully connected layer uses a SoftMax layer to calculate the probabilities for the four immature cells (Figure 3).
c. Residual network:

Residual Networks (ResNets) are type of CNN [47]. It was developed to enhance the accuracy of networks by increasing their depth, while addressing the issue of vanishing gradients. The process of constructing these networks involves sequentially stacking residual blocks and incorporating shortcut connections. The shortcut connection is a mathematical function that maintains the original input and links it to an activation function at an earlier stage in the network. This method is particularly useful when the activation of a certain layer tends to diminish toward zero as the network deepens. This study involved the adaptation and fine-tuning of ResNet50 to extract features from image embeddings with dimensions of 112 × 112 × 8, which deviated from the standard input configuration of (224, 224, 3) (Figure 4).

The mathematical representation of this process is as follows:

(7) $$a^{i+3}=f\;\left(y^{i+3}+a^{i}\right)$$

where

(8) $$y^{i+3}=w^{i+3}*c^{i+3}+b^{i+3}$$

By substituting (8) into (7), we obtain the following:

(9) $$a^{i+3}=f\left(w^{i+3}*c^{i+3}+b^{i+3}+a^{i}\right)$$

Assuming that f is a ReLU function, we obtain the following:

(10) $$a^{i+3}=\left\{ \;0\begin{array}{c} w^{i+3}*c^{i+3}+b^{i+3}+a^{i}\;\;\;\;\;\;\;\;\;\;\;\;\;\;\text{if}\;w^{i+3}*c^{i+3}+b^{i+3}+a^{i\;}\;>\;0\\ \;\;\;\;\;\;\;\;\;\;\;\;\;\;\;\;\;otherwise\\ \;\;\;\;\;\;\;\;\;\;\;\;\;\;\;\;\;\;\;\;\;\;\;\;\; \end{array}\right.$$

If the activation function for layer i + 3 ($a^{i+3})$ approaches zero, then

$$c^{i+3}=0\;\text{and}\;b^{i+3}=0$$

Therefore,

(11) $$a^{i+3}=\left\{ \begin{array}{c} a\;\;\;\;\;\;\;\;\;\;\;\;\;\;\;\;\text{if}\;a^{i\;}\;>\;0\;\\ 0\;\;\;\;\;\;\;\;\;\;\;\;\;\;\mathrm{otherwise}\; \end{array}\right.$$

The ResNet architecture adheres to two fundamental design principles:

1. The total number of filters in each layer is determined by the dimensions of the resulting feature map.
2. When the size of the feature map is reduced by half, the number of filters is increased two fold to preserve the time complexity of each layer.

This study uses ResNet50, a specific variant of ResNets, for feature extraction. The comprises 50 layers, including 48 convolutional layers, 1 MaxPool layer, and 1 average pool layer. The architecture employs a bottleneck design by using 1×1 convolutions, which substantially reduces the number of parameters and matrix multiplications required. This accelerates the training of individual layers. The ResNet50 architecture comprises the following components:

• A 7 × 7 convolutional kernels, a stride of 2, and 64 filters
• A 3 × 3 max pooling with a stride of 2
• Three sets of repeating blocks with each block comprising three consecutive convolutional layers, resulting in **nine convolutional layers,** as follows:
  o 3 × 3 kernel and 64 filters
  o 1 × 1 kernel and 64 filters
  o 1 × 1 kernel and 256 filters
• Four sets of three repeating blocks, with each block comprising three consecutive convolutional layers, resulting in **12 convolutional layers,** as follows:
  o 1 × 1,128 filters
  o 3 × 3,128 filters
  o 1 × 1,512 filters
• Six sets of repeating blocks with each block comprising three consecutive convolutional layers, resulting in **18 convolutional layers,** as follows:
  o 1 × 1, 256 filters
  o 3 × 3,256 filters
  o 1 × 1024 filters
• Three sets of repeating blocks with each block comprising three consecutive convolutional layers, resulting in **nine convolutional layers,** as follows:
  o 1 × 1,512 filters
  o 3 × 3,512 filters
  o 1 × 2048 filters
  o Average pooling, followed by a fully connected layer containing **four nodes** and a SoftMax activation function.

**Phase III: Classification**

The classification phase was performed using a weight ensemble of two MLPs, each consisting of a fully connected layer of 1,000 neurons. The optimal weights were determined by a grid search of w between 0.1 and 0.5 (Figure1).

### 3.3 Model training

The dataset was divided into 80/20 splits for training and validation. Stochastic gradient descent with a momentum of 0.8 and a learning rate of 0.00001 was employed during model training. For binary classification, a single neuron and a sigmoid activation function were used to classify mature and immature WBCs. For multiclassification, the model incorporated a dense layer of eight neurons using the SoftMax loss function to classify WBCs into eight distinct subtypes. To train the autoencoder, we used the Adam optimizer with a learning rate of 0.0005 and the MSE as the loss function. The ImageDataGenerator class was used to generate image batches from a physical directory to improve memory allocation. The model was constructed using a computational setup featuring an Intel® Core™ i7-9750H CPU running at 2.60 GHz with 192 cores, 16 GB of RAM, and equipped with an NVIDIA GeForce RTX 2070 GPU with max design capabilities. The algorithm was implemented in Python, leveraging Keras along with additional image-processing libraries to extract manually engineered features.

**Phase Iv: Validation**

The classification model’s performance was assessed based on the validation dataset, using the following assessment criteria: overall accuracy, precision, sensitivity, F-score, and area under the receiver operating characteristic curve (AUC). The assessment of the synthetic images generated by the CAE involved a comparison of the similarity between the original and the synthetic images using the MSE metric.

## 4 Results and discussion

This section provides a comprehensive analysis of the results obtained by implementing the proposed model. The phase results, comparative analysis, and implications for the study objectives are presented.

• **Phase I: Preprocessing**

The preprocessing phase involves the segmentation of WBCs and correction of the imbalance distribution of WBCs. The image segmentation process was performed by employing the CMYK-Moment's localization region of interest extraction and CNN feature fusion method [25]. The process of segmenting WBCs generates a context-free framework, that enables the identification and extraction of independent features associated with WBCs.

**Figure 5:**
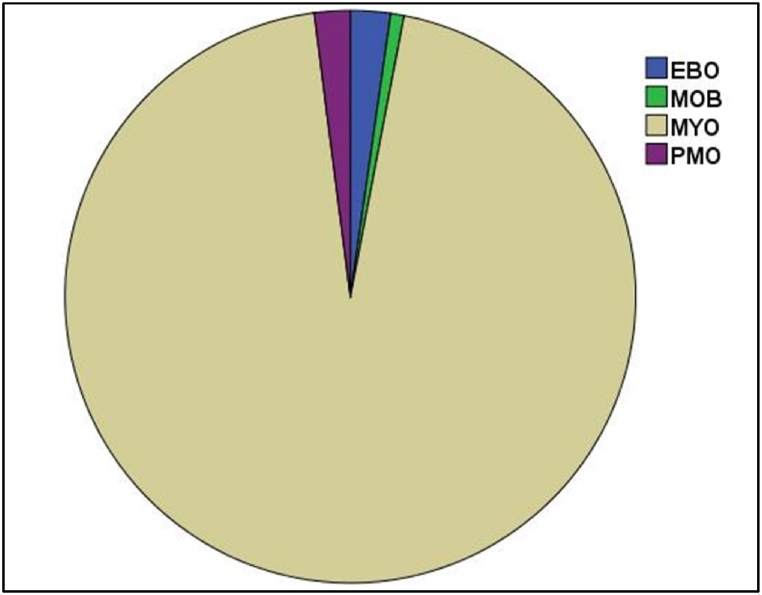
Distribution of immature WBCs in the dataset.

Data augmentation was used to mitigate the problem of imbalanced data distribution and enhance the capability of the feature extraction model to effectively learn significant features from the minority classes, namely EBO, MOB, and PMO. Figure 5 shows the initial distribution of immature WBCs before data augmentation. Data analysis demonstrated a predominant Myeloblast classification (95.5%), with the remaining three categories collectively accounting for only 4.5%. After data augmentation, the dataset was expanded to 40,000 images, ensuring an equal distribution of 10,000 images per subtype. This balanced dataset facilitated subsequent feature extraction and classification tasks.

• **Phase II: Feature extraction**
  o **Images embedding representation.**

**Figure 6:**
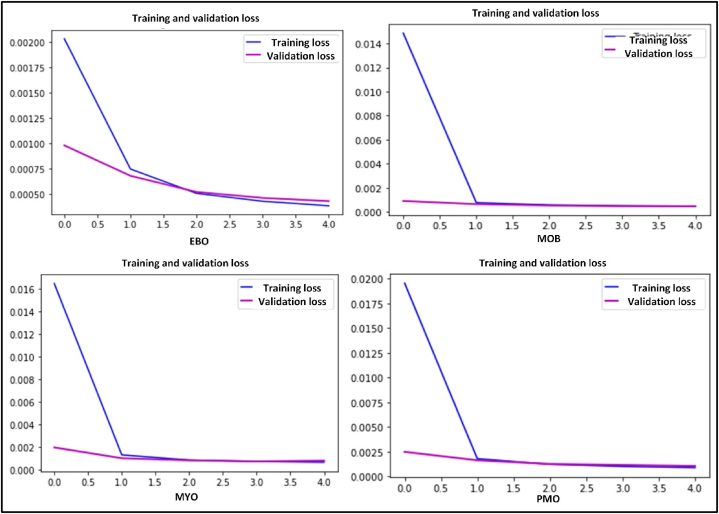
CAE learning curves of immature WBCs.

**Figure 7:**
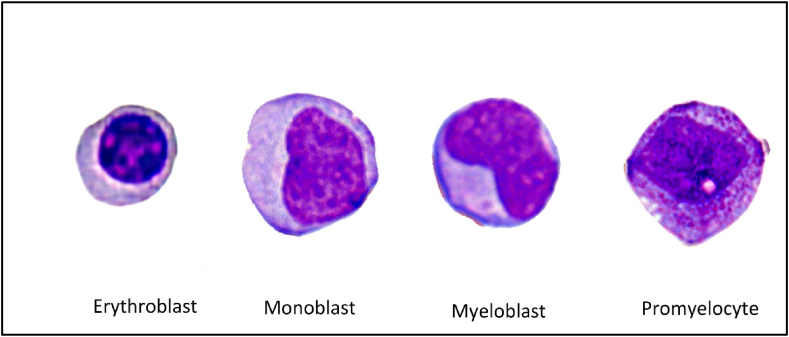
Examples of synthetic images generated by the DCAE model.

Four GT-CAE models were used to generate four subtypes of immature WBCs, each with 10,000 images. The MSE across models ranged from 0.001 to 0.002, indicating high fidelity between the original and generated images, underscoring the models’ effectiveness in replicating original images. Figures 6 and 7 show the CAE learning curves and synthetic image examples generated by the CAE model, respectively. Image embedding was obtained by resizing original images to 112 × 112 × 8 using the encoder unit.

**Figure 8:**
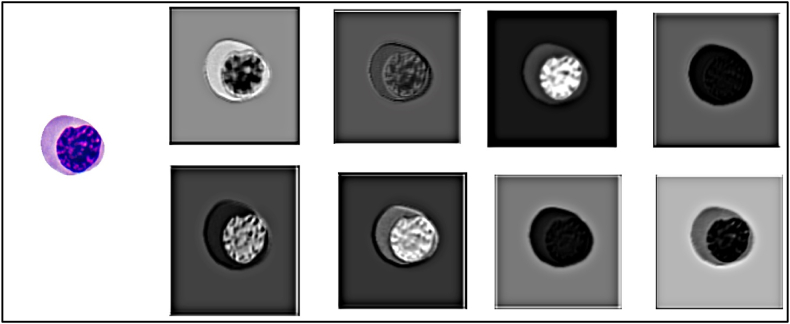
Eight distinct instances of EBO embedding representation using the proposed DCAE model.

Figure 8 shows reduced image representations (embeddings) of EBO. This study further explored these new image representations as inputs to the VGG19 and ResNet models.

o **Transfer-learning.**
  ⁃ **VGG19**
    - **Phase1: Mature versus. immature binary classification**

**Figure 9:**
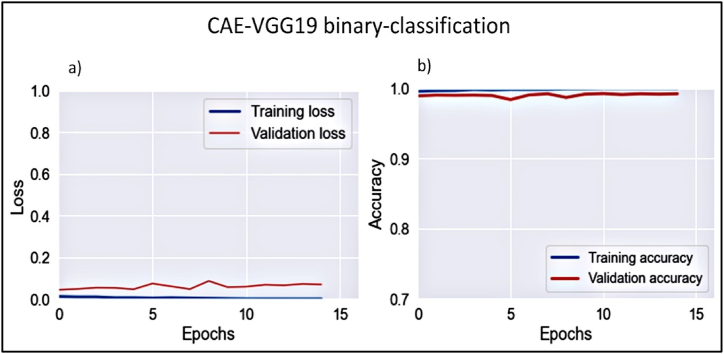
Learning curves of CAE-VGG19 binary classification.

In this study phase, WBCs were classified according to maturity into mature and immature groups. The curated dataset comprised 21,600 images, equally divided into 10,800 images, equally devided into mature and immature WBCs. The model exhibited optimal convergence, as evidenced by declining loss and increasing accuracy for both the training and validation learning curves ,Figure 9 (a, b).

The model achieved a sensitivity of 99.2%, precision of 98%, and overall accuracy of 99.3%. The model’s capacity to differentiate between mature and immature WBCs was confirmed by an AUC value of 99.2%.

- **Phase II: The immature WBC multiclassification model**

**Figure 10:**
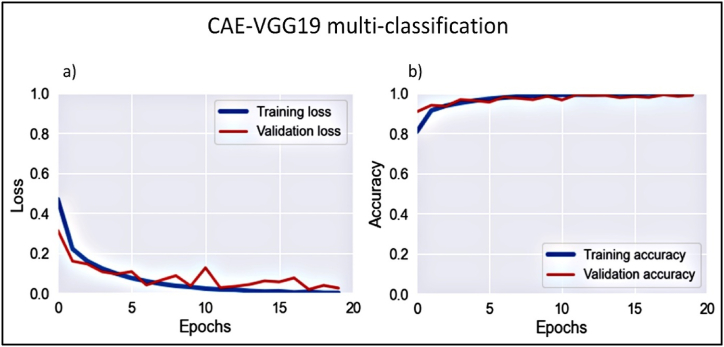
Learning curves of CAE-VGG19 multi-classification of immature WBCs.

This phase classified immature WBCs into four distinct classes, as previously described. The model stability was assessed via a convergence analysis using the training and validation learning curves. Figure 10 (a, b) shows these curves in terms of loss and overall accuracy.

Data from Figure 10 indicates robust convergence from the second epoch onward. The performance of the multiclassification model was assessed by comparing the pathologist-provided ground-truth labels with the model’s predictions. The vector $\Phi =\left[\Phi_{1},\Phi_{2},\Phi_{3},\Phi_{4}\right]$ denotes the model’s predictions for each, where $\Phi_{i}$ reprents the probability of the $i^{th}$ subclass, with i ∈ [0,1,2,3].

The Argmax function determines the class with the highest predicted probability. The model evaluation used accuracy, sensitivity, F-score, and AUC metrics. Precision and sensitivity values for each class are given in Table 3.

**Table 3:** CAE-VGG19 multiclassification overall and class-wise performance measures.

	Precision	Sensitivity	F-score
*EBO*	0.999	0.999	0.999
*MOB*	0.999	0.750	0.857
*MYO*	0.989	0.999	0.994
*PMO*	0.999	0.705	0.827
*Overall accuracy*	0.989		
*AUC*	0.999		

The study findings revealed that the model achieved high accuracy in classifying immature WBCs, particularly myeloblasts, which are crucial for diagnosing acute AML, with an overall accuracy of 98.9%. It demonstrated superior precision (98.9%) and sensitivity (99.9%) for detecting myeloblasts. In addition, the model showed excellent precision (99.9%) in identifying erythroblasts, monoblasts, and promyelocytes, although the sensitivity for monoblasts and promyelocytes was lower, at (**75% and 70% respectively).**

**Figure 11:**
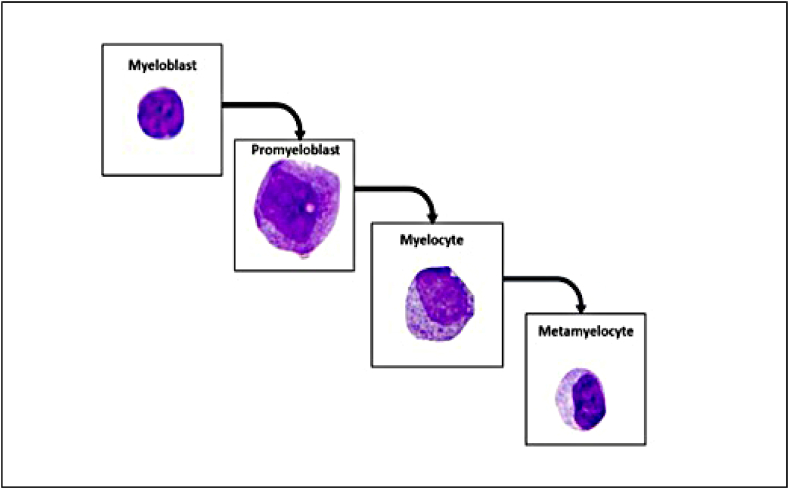
Stages of myelopoiesis.

The reduced sensitivity for monoblast and promyelocyte detection may be due to their limited presence in the validation dataset. Figure 15 (a) shows that monoblasts and promyelocytes are often misclassified as myeloblasts. This misclassification likely arises from morphological overlaps with myeloblasts, such as similar nuclear-to-cytoplasmic ratios, nucleus shapes, cytoplasm distributions of monoblasts, and the morphological and developmental proximity of promyelocytes to myeloblasts during myelopoiesis (Figure 11).

The model showed excellent performance in accurately classifying erythroblasts and promyelocytes, achieving precision rates of 99.9% and 99.9%, respectively. In addition, the model demonstrated sensitivity rates of 99.9% for erythroblasts and 70.5% for promyelocytes.

⁃ **ResNet50**
  - **Phase1: Mature versus. immature binary classification**

**Figure 12:**
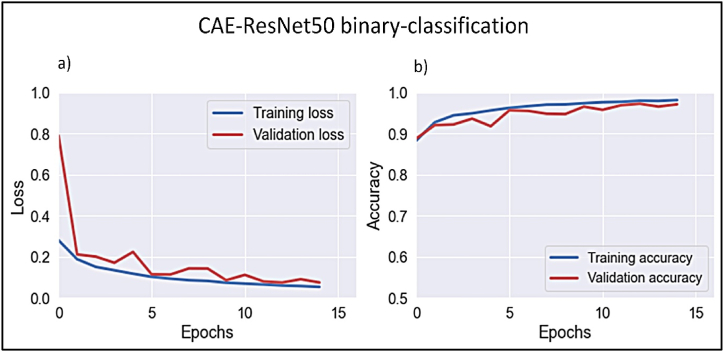
Learning curves for CAE-ResNet binary classification model.

ResNet50 exhibited an overall WBC classification accuracy of 97.1% for mature versus immature categories, with a sensitivity of 97.3% and precision of 97.1%. The model demonstrated strong discriminatory capability, as evidenced by an AUC of 99.5%. Moreover, the model achieved excellent convergence, as demonstrated by consistent decreases in loss and improvements in accuracy throughout the training and validation phases, Figure 12 (a, b).

- **Phase II: Immature WBC multiclassification model**

**Figure 13:**
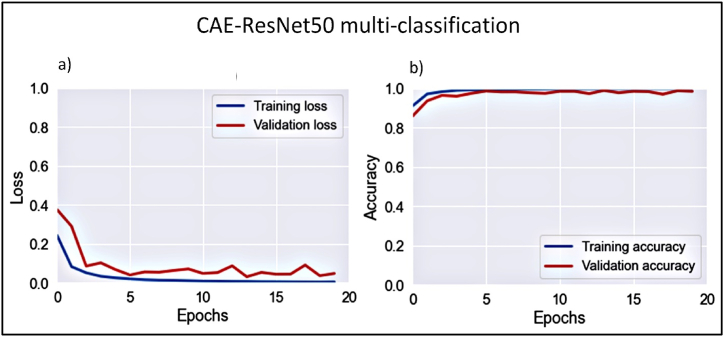
Learning curves of CAE-ResNet multi-classification model.

Using ResNet, the proposed model exhibited convergence following five epochs, as evidenced by the training and validation learning curves. Figure 13 (a, b) shows these curves, detailing loss and overall accuracy, with convergence beginning after the fifth epoch.

The ResNet model obtained a classification accuracy of 98.5% across all categories. The myeloblast exhibited outstanding performance, with precision and sensitivity rates of 98.6% and 99.8%, respectively. Conversely, the promyelocytes exhibited the least sensitive performance, with a sensitivity of 57.1% and a higher rate of false-positive results compared with CAE-VGG19. Furthermore, erythroblast demonstrated a reduced level of precision compared with the other classes, with a sensitivity rate of 93.3%, which also suggests a higher occurrence of false-negative results compared to CAE-VGG19. Figure 15(b) reveals that some myeloblasts were misclassified as erythroblasts because of their morphological similarities, notably the difference in size between the nucleus and cytoplasm. In addition, both erythroblasts and myeloblasts exhibited significant variations in nuclear and cytoplasmic contents. The comparative assessment indicated that the CAE-ResNet and CAE-VGG19 models performed similarly, albeit with reduced precision for erythroblast identification and lower sensitivity for promyelocyte detection. Table 4 shows the precision and sensitivity for each class as determined by CAE-ResNet.

• **Phase III: Classification**

**Table 4:** CAE-ResNet multiclassification overall and class-wise performance measures

	Precision	Sensitivity	F-score
*EBO*	0.933	0.999	0.965
*MOB*	0.999	0.999	0.999
*MYO*	0.986	0.998	0.992
*PMO*	0.999	0.571	0.9600
*Overall accuracy*	0.985		
*AUC*	0.999		

**Figure 14:**
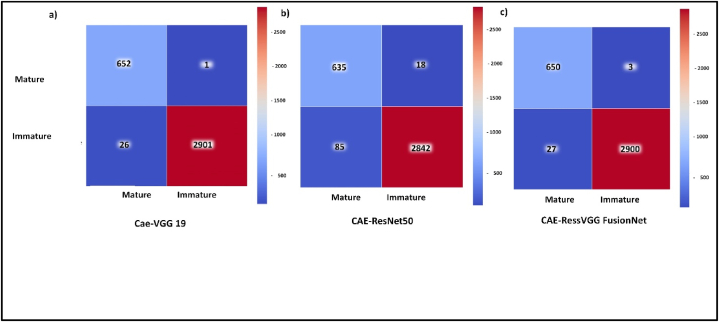
a) CAE-VGG19, b) CAE-ResNet, c) CAE-ResVGG FusionNet confusion matrix for binary classification of mature versus immature WBCs.

Classification involved the integration of CAE-VGG19 and CAE-ResNet via an ensemble approach, encompassing both binary classification and multiclassification. The binary classification model achieved an overall accuracy of 99.2%, precision of 99.1%, sensitivity of99.2% and AUC of 99.7%. Figure 14 (a-c) shows the confusion matrix for CAE-VGG19, CAE-ResNet, and CAE-ResVGG FusionNet, respectively.

**Figure 15:**
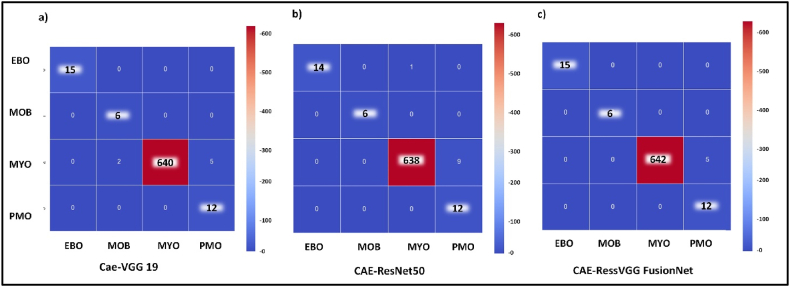
a) CAE-VGG19, b) CAE-ResNet, c) CAE-ResVGG FusionNet multi-classification confusion matrix.

For multiclassification, the integrated model enhanced overall accuracy, AUC, class-specific precision, and sensitivity, Figure (a-c). The outcomes of multiclassification of the integrated models are detailed in Table5, and Figure 15 (c) shows the confusion matrix illustrating the performance of model integration.

**Table 5:** CAE-ResVGG FusionNet overall and class-wise performance measures.

	Precision	Sensitivity	F-score
EBO	0.999	0.999	0.999
MOB	0.999	0.999	0.999
MYO	0.992	0.999	0.996
PMO	0.999	0.70	0.827
Overall accuracy	0.9956		
AUC	0.999		

The results of the proposed model were compared with those of existing classification studies on the same dataset, revealing significant enhancements in the overall and class-specific performance metrics such as sensitivity, precision, overall accuracy, and AUC. The model effectively addresses the gaps identified in previous studies by leveraging DL features that are capable of capturing complex and concealed patterns in immature WBC images. This approach distinguishes it from Dasariraju et al.[33] and Dincic et al. [34], which relies on handcrafted features governed by human-defined rules that have inherent limitations when capturing intricate features [43]. The proposed model integrates WBC segmentation into the classification framework using the CMYK-moment localization and a CNN feature fusion model, which was developed by Elhassan et al. [25]. In contrast, Matek et al., extracted the features of WBCs and classified them from the images without segmentation. The integration of segmentation improved the model’s ability to extract generalized features of WBCs in a contextually independent manner. The effectiveness of our approach is underscored by superior results compared to Matek et al., who did not utilize WBC segmentation. In addition, results obtained by DL models developed by Bairaboina et al.[36] and Elhassan et al. [22], who incorporated segmentation into their classification frameworks, demonstrated improved classification accuracy with those of Matek et al. [3]. The proposed model leveraged the strengths of these previous approaches by incorporating WBC segmentation and DL-based features into a hybrid model that integrates a deep CAE, VGG19, and ResNet to segment WBCs and extract features. This highlights the effectiveness of the proposed framework in terms of enhancing classification accuracy using robust segmentation techniques. Table 6 presents the performance improvement of the proposed model compared to previous studies. However, it should be noted that the proposed model did not achieve higher sensitivity for PMO compared with the findings of Bairaboina et al. [36] and Dincic et al.[34]. The proposed model achieved a sensitivity of 69.8%, whereas Bairaboina et al. [36] and Dincic et al.[34] reported sensitivities of 98.3% and 71%, respectively.

**Table 6:** Adjusted class-wise probabilities of immature WBCs.

	Matek et al. (2019)		Dasariraju et al. (2020)		Dincic et al. (2021)		Elhassan et al. (2023)		Bairaboina and Battula (2023)		CAE-ResVGG FusionNet	
Cells	Precision	Sensitivity	Precision	Sensitivity	Precision	Sensitivity	Precision	Sensitivity	Precision	Sensitivity	Precision	Sensitivity
EBO	0.7500	0.8700	0.9123	0.8710	0.8600	1.0000	0.9679	0.9303	0.9973	0.9867	**0.990**	**0.990**
MOB	0.5200	0.5800	0.7982	0.9540	0.8800	0.9600	0.9679	0.8512	0.9849	0.9847	**0.990**	**0.990**
MYO	0.9400	0.9400	0.8826	0.9009	0.8000	0.9600	0.9582	0.9798	0.995	0.995	**0.983**	**0.990**
PMO	0.6300	0.5400	0.5701	0.5439	0.8900	0.7100	0.6484	0.5245	0.9826	0.983	**0.990**	**0.698**
Overall accuracy	Not calculated		0.8676		0.81		0.9312		98.61		**0.999**	
AUC	0.986		Not calculated		Not calculated		0.9897		Not calculated		**0.9964**	

## 5 Conclusion

This study presents a DL framework, called “CAE-ResVGG FusionNet” for AML diagnosis by analyzing WBCs, thereby highlighting the potential of AI in medical diagnostics. By classifying WBCs into mature and immature types and further into specific subtypes, the model demonstrated superior accuracy, specificity, and sensitivity, highlighting its ability to expedite diagnosis and treatment decisions. Such advances are crucial for conditions such as AML, in which early detection can significantly improve patient outcomes.

Leveraging AI technologies, such as CAE-ResVGG FusionNet can enhance the precision of diagnoses for different types of blood cancers and contagious illnesses, potentially resulting in early identification and tailored treatment approaches.

The proposed model’s ability to identify complex patterns in disease data underscores its potential to discover novel biomarkers and disease etiologies, thereby enabling targeted therapeutic interventions and precision medicine approaches.

Furthermore, the implementation of AI frameworks in clinical settings can improve the workflow efficiency, reduce diagnostic errors, optimize the allocation of resources, and ultimately enhance the quality of healthcare delivery and patient care.

## 6 Future work and limitations

This study faced limitations, including a skewed distribution of immature WBCs, which were addressed partially using data augmentation techniques, however, the original data quality could not be fully replicated. The challenge of AI interpretability remains, highlighting the need for improved transparency and reliability in AI diagnostics. Future research may prioritize the development of new datasets and improve existing datasets by increasing and diversifying them to enhance model generalizability. In addition, future research focuses on enhancing model performance by incorporating real-time patient data streams into the AI frameworks, which can significantly improve diagnostic accuracy and prediction capacities. Furthermore, further research should prioritize the integration of AI models into clinical practice and the establishment of policies to effectively address concerns regarding privacy, patient consent, and algorithmic bias. This is crucial for maintaining trust and dependability in AI-driven diagnostics.

## Data availability

The dataset utilized in this study is available under The Cancer Imaging Archive (TCIA): “Matek, C., Schwarz, S., Marr, C., & Spiekermann, K. (2019). A Single-cell Morphological Dataset of Leukocytes from AML Patients and Non-malignant Controls [Data set]. The Cancer Imaging Archive. https://doi.org/10.7937/tcia.2019.36f5o9ld“.

Mahmoud Aljurf: Writing – review & editing, Project administration, Investigation. Esmaeil ahmed mohamed elhassan ahmed: Writing – review & editing, Visualization, Validation, Investigation. Yura Mohd Elkamali: Resources, Investigation. Abdulalem ALI Ali: Writing – review & editing, Validation, Project administration. Siti Zaiton Mohd Hashim: Writing – review & editing, Validation, Supervision, Project administration, Investigation. Mohd Shafry Mohd Rahim: Writing – review & editing, Visualization, Validation, Project administration, Investigation, Conceptualization. Ahmed Hamza Osman: Writing – review & editing, Validation, Project administration, Investigation. Tusneem Ahmed Mohamed Elhassan: Writing – review & editing, Writing – original draft, Visualization, Validation, Software, Resources, Project administration, Methodology, Investigation, Formal analysis, Data curation, Conceptualization

## Declaration of Competing Interest

☒ The authors declare that they have no known competing financial interests or personal relationships that could have appeared to influence the work reported in this paper.

☐ The authors declare the following financial interests/personal relationships which may be considered as potential competing interests:

## References

[1] Bigorra L. (2017). Feature analysis and automatic identification of leukemic lineage blast cells and reactive lymphoid cells from peripheral blood cell images. Journal of clinical laboratory analysis.

[2] Dulaimi K.A.-. (2021). Segmentation of White Blood Cell, Nucleus and Cytoplasm in Digital Haematology Microscope Images: A Review–Challenges, Current and Future Potential Techniques. IEEE Reviews in Biomedical Engineering.

[3] Matek C. (2019). Human-level recognition of blast cells in acute myeloid leukaemia with convolutional neural networks. Nature Machine Intelligence.

[4] Bigorra, L., et al., Feature analysis and automatic identification of leukemic lineage blast cells and reactive lymphoid cells from peripheral blood cell images*.* 2017. 31(2): p. e22024. 10.1002/jcla.22024 PMC6817297 27427422

[5] Han D. (2023). LMCA: a lightweight anomaly network traffic detection model integrating adjusted mobilenet and coordinate attention mechanism for IoT. Telecommunication Systems.

[6] Wang H. (2023). NAS-YOLOX: a SAR ship detection using neural architecture search and multi-scale attention. Connection Science.

[7] Lakshmanna, K., et al., Improved metaheuristic-driven energy-aware cluster-based routing scheme for IoT-assisted wireless sensor networks*.* 2022. 14(13): p. 7712.

[8] Abd Algani, Y.M., et al., Leaf disease identification and classification using optimized deep learning*.* 2023. 25: p. 100643.

[9] Hartmeier, P., et al., Tracking garnet dissolution kinetics in 3D using deep learning grain shape classification*.* 2024. 65(3): p. egae005.

[10] Aamir, M., et al., AMDDLmodel: Android smartphones malware detection using deep learning model*.* 2024. 19(1): p. e0296722. 10.1371/journal.pone.0296722 PMC10798489 38241330

[11] Ibrahim, A.U., et al., Pneumonia classification using deep learning from chest X-ray images during COVID-19*.* 2021: p. 1-13. 10.1007/s12559-020-09787-5 PMC7781428 33425044

[12] Saleh, A., R. Sukaik, and S.S. Abu-Naser. Brain Tumor Classification Using Deep Learning. in 2020 International Conference on Assistive and Rehabilitation Technologies (iCareTech). 2020.

[13] Sharif M.I. (2022). A decision support system for multimodal brain tumor classification using deep learning. Complex & Intelligent Systems.

[14] Wahlang, I., et al., Brain magnetic resonance imaging classification using deep learning architectures with gender and age*.* 2022. 22(5): p. 1766. 10.3390/s22051766 PMC8914787 35270913

[15] Mohan, P., et al., Handcrafted deep-feature-based brain tumor detection and classification using mri images*.* 2022. 11(24): p. 4178.

[16] Menaouer, B., et al., Diabetic retinopathy classification using hybrid deep learning approach*.* 2022. 3(5): p. 357.

[17] Guleria, K., et al., Early prediction of hypothyroidism and multiclass classification using predictive machine learning and deep learning*.* 2022. 24: p. 100482.

[18] Joshi A.A., Aziz R.M. (2024). Deep learning approach for brain tumor classification using metaheuristic optimization with gene expression data. J.I.J.o.I.S. and Technology.

[19] İncir R., Bozkurt F. (2024). A study on effective data preprocessing and augmentation method in diabetic retinopathy classification using pre-trained deep learning approaches. J.M.T. and Applications.

[20] Vakiti A and M. P. Acute Myeloid Leukemia*.* . 2023; Available from: https://www.ncbi.nlm.nih.gov/books/NBK507875/.

[21] Krappe, S., et al. Automated morphological analysis of bone marrow cells in microscopic images for diagnosis of leukemia: Nucleus-plasma separation and cell classification using a hierarchical tree model of hematopoesis. in Medical Imaging 2016: Computer-Aided Diagnosis. 2016. International Society for Optics and Photonics.

[22] Elhassan T.A. (2023). Classification of Atypical White Blood Cells in Acute Myeloid Leukemia Using a Two-Stage Hybrid Model Based on Deep Convolutional Autoencoder and Deep Convolutional Neural Network. J Diagnostics.

[23] Dinčić M. (2021). Morphological, fractal, and textural features for the blood cell classification: the case of acute myeloid leukemia. Eur Biophys J.

[24] Choi J.W. (2017). White blood cell differential count of maturation stages in bone marrow smear using dual-stage convolutional neural networks. PloS one.

[25] Elhassan T.A.M. (2022). Feature Extraction of White Blood Cells Using CMYK-Moment Localization and Deep Learning in Acute Myeloid Leukemia Blood Smear Microscopic Images. IEEE Access.

[26] Walker, H.K., W.D. Hall, and J.W. Hurst, in Clinical Methods: The History, Physical, and Laboratory Examinations. 1990, Butterworths Copyright © 1990, Butterworth Publishers, a division of Reed Publishing.: Boston. 21250045

[27] Kazemi F., Najafabadi T.A., Araabi B.N. (2016). Automatic Recognition of Acute Myelogenous Leukemia in Blood Microscopic Images Using K-means Clustering and Support Vector Machine. J Med Signals Sens.

[28] Suryani E. (2017). Classification of Acute Myelogenous Leukemia (AML M2 and AML M3) using Momentum Back Propagation from Watershed Distance Transform Segmented Images. Journal of Physics: Conference Series.

[29] Wiharto, E.S., et al. Cells identification of acute myeloid leukemia AML M0 and AML M1 using K-nearest neighbour based on morphological images. in 2017 International Conference on Data and Software Engineering (ICoDSE). 2017. IEEE.

[30] Wiharto W., Suryani E., Putra Y.R. (2019). Classification of blast cell type on acute myeloid leukemia (AML) based on image morphology of white blood cells. J TELKOMNIKA.

[31] Harjoko, A., et al. Classification of acute myeloid leukemia subtypes M1, M2 and M3 using active contour without edge segmentation and momentum backpropagation artificial neural network. in MATEC Web of Conferences. 2018. EDP Sciences.

[32] Bigorra L. (2017). Feature Analysis and Automatic Identification of Leukemic Lineage Blast Cells and Reactive Lymphoid Cells from Peripheral Blood Cell Images. J Clin Lab Anal.

[33] Dasariraju S., Huo M., McCalla S. (2020). Detection and Classification of Immature Leukocytes for Diagnosis of Acute Myeloid Leukemia Using Random Forest Algorithm. Bioengineering-Basel.

[34] Dincic M. (2021). Morphological, fractal, and textural features for the blood cell classification: the case of acute myeloid leukemia. European Biophysics Journal with Biophysics Letters.

[35] Elhassan, T.A., et al., Classification of atypical white blood cells in acute myeloid leukemia using a two-stage hybrid model based on deep convolutional autoencoder and deep convolutional neural network*.* 2023. 13(2): p. 196. 10.3390/diagnostics13020196 PMC9858290 36673006

[36] Bairaboina, S.S.R. and S.R. Battula, Ghost-ResNeXt: An Effective Deep Learning Based on Mature and Immature WBC Classification*.* 2023. 13(6): p. 4054.

[37] Hegde R.B. (2019). Feature extraction using traditional image processing and convolutional neural network methods to classify white blood cells: a study. Australasian Physical & Engineering Sciences in Medicine.

[38] Tareef, A., et al. Automated multi-stage segmentation of white blood cells via optimizing color processing. in 2017 IEEE 14th international symposium on Biomedical imaging (ISBI 2017). 2017. IEEE.

[39] Al-Dulaimi, K., et al. Classification of white blood cell types from microscope images: Techniques and challenges. 2018.

[40] Setiawan, A., et al. Classification of cell types in Acute Myeloid Leukemia (AML) of M4, M5 and M7 subtypes with support vector machine classifier. in 2018 International Conference on Information and Communications Technology (ICOIACT). 2018.

[41] Dinčić M. (2021). Morphological, fractal, and textural features for the blood cell classification: the case of acute myeloid leukemia. European Biophysics Journal.

[42] Qin, F., et al., Fine-grained leukocyte classification with deep residual learning for microscopic images*.* 2018. 162: p. 243-252. 10.1016/j.cmpb.2018.05.024 29903491

[43] Elhassan, T.A., et al., Segmentation of White Blood Cells in Acute Myeloid Leukemia Microscopic Images: A Review, in Prognostic Models in Healthcare: AI and Statistical Approaches, T. Saba, A. Rehman, and S. Roy, Editors. 2022, Springer Nature Singapore: Singapore. p. 1-24.

[44] Matek C. (2019). A Single-cell Morphological Dataset of Leukocytes from AML Patients and Non-malignant Controls. The Cancer Imaging Archive.

[45] Masci, J., et al. Stacked convolutional auto-encoders for hierarchical feature extraction. in Artificial Neural Networks and Machine Learning–ICANN 2011: 21st International Conference on Artificial Neural Networks, Espoo, Finland, June 14-17, 2011, Proceedings, Part I 21. 2011. Springer.

[46] Simonyan K., Zisserman A. (2014). Very deep convolutional networks for large-scale image recognition. J.a.p.a..

[47] Xie, S., et al. Aggregated residual transformations for deep neural networks. in Proceedings of the IEEE conference on computer vision and pattern recognition. 2017.

